# Endplate volumetric bone mineral density biomechanically matched interbody cage

**DOI:** 10.3389/fbioe.2022.1075574

**Published:** 2022-12-06

**Authors:** Yuanzhi Weng, Mingyuan Di, Tianchi Wu, Xinlong Ma, Qiang Yang, Weijia William Lu

**Affiliations:** ^1^ Department of Orthopaedics and Traumatology, Li Ka Shing Faculty of Medicine, The University of Hong Kong, Hong Kong, Hong Kong SAR, China; ^2^ Department of Orthopaedics and Traumatology, The University of Hong Kong-Shenzhen Hospital, Shenzhen, China; ^3^ Graduate School, Tianjin Medical University, Tianjin, China; ^4^ Department of Spine Surgery, Tianjin Hospital, Tianjin University, Tianjin, China; ^5^ Tianjin Hospital, Tianjin University, Tianjin, China; ^6^ Research Center for Human Tissue and Organs Degeneration, Institute of Biomedicine and Biotechnology, Shenzhen Institute of Advanced Technology, Chinese Academy of Sciences (CAS), Shenzhen, China

**Keywords:** interbody fusion, cage subsidence, endplate, porous cage design, patient-specific customization implant

## Abstract

Disc degenerative problems affect the aging population, globally, and interbody fusion is a crucial surgical treatment. The interbody cage is the critical implant in interbody fusion surgery; however, its subsidence risk becomes a remarkable clinical complication. Cage subsidence is caused due to a mismatch of material properties between the bone and implant, specifically, the higher elastic modulus of the cage relative to that of the spinal segments, inducing subsidence. Our recent observation has demonstrated that endplate volumetric bone mineral density (EP-vBMD) measured through the greatest cortex-occupied 1.25-mm height region of interest, using automatic phantomless quantitative computed tomography scanning, could be an independent cage subsidence predictor and a tool for cage selection instruction. Porous design on the metallic cage is a trend in interbody fusion devices as it provides a solution to the subsidence problem. Moreover, the superior osseointegration effect of the metallic cage, like the titanium alloy cage, is retained. Patient-specific customization of porous metallic cages based on the greatest subsidence-related EP-vBMD may be a good modification for the cage design as it can achieve biomechanical matching with the contacting bone tissue. We proposed a novel perspective on porous metallic cages by customizing the elastic modulus of porous metallic cages by modifying its porosity according to endplate elastic modulus calculated from EP-vBMD. A three-grade porosity customization strategy was introduced, and direct porosity-modulus customization was also available depending on the patient’s or doctor’s discretion.

## Introduction

Disc degenerative disease (DDD) is a global concern owing to an increasingly aging population. DDD is evitable as it is caused mainly by aging and fatigue (Battié and Videman, 2006). Effective treatment for DDD is discectomy wherein the disc is partly removed as there is an extra abnormal region. After discectomy, DDD is finally cured through interbody fusion (IF), a surgical process connecting two vertebrae permanently by implanting a cage between them (Mobbs et al., 2015). Herein, the cage contributes to intervertebral decompression and stability, thus promoting optimal vertebral fusion (Jain et al., 2016).

We designed a biomechanically matched cage based on volumetric bone mineral density (vBMD) transferred elastic modulus, obtained through quantitative computed tomography (QCT) scanning. Unlike DXA, QCT has the benefits of higher accuracy and reproductivity (Li et al., 2014). Another benefit of this patient-specific cage customization is the validated phantomless QCT system (Liu et al., 2022) used for endplate vBMD (EP-vBMD) measurements.

## Interbody fusion surgery and degenerative disc disease

According to a study in 2018, 5.5% of the world’s population (over 400 million people) suffers from DDD, the most frequent spinal degenerative disease relative to others, including spondylolisthesis and stenosis (Ravindra et al., 2018). A study focused on DDD in Japanese (n = 975) indicated that people aged over 50 years have a high incidence rate of DDD (over 90%) (Teraguchi et al., 2014). In recent years, IF as a main surgical treatment for DDD is gaining increasing popularity, worldwide; a remarkable example is the UK wherein IF cases increased by over 60% from 2005 to 2015 (Provaggi et al., 2018; Reisener et al., 2020).

IF surgeries can be performed on different sections of the human spine including the cervical, thoracic, and lumbar regions. The prevalence rate of DDD for the lumbar spine L4∼L5 is the highest in comparison to those for the thoracic and cervical regions (Teraguchi et al., 2014). Coordinate surgery for lumbar DDD, lumbar IF (LIF), can be classified as anterior LIF (ALIF), posterior LIF (PLIF), lateral LIF (LLIF), transforaminal LIF (TLIF), and oblique LIF (OLIF) (Mobbs et al., 2015). The LIF type determines cage design including size and shape as these are needed to meet surgical requirements like implant channel width or specific surgical instrumentation design, affecting the operational risks of fusion procedures.

## Subsidence risk of interbody fusion

LIF poses intraoperative and postoperative risks. Intraoperative risks include dangerous issues for surgical processes like potential muscle injury, while postoperative risks include hazards after surgery like cage subsidence.

TLIF, first achieved in the 1980s, has obvious advantages owing to the intraoperative aspect. The nerve roots, ureter/blood vessels, lumbar plexus nerve, and sympathetic nerves do not need to be considered in TLIF, unlike in PLIF, ALIF, XLIF, and OLIF respectively (Mobbs et al., 2015). TLIF can also be achieved through the minimally invasive path, resulting in benefits including less blood loss (Lener et al., 2020). The relatively lower risk and clinical benefits of TLIF indicate its great potential in dominant LIF surgeries.

However, TLIF has a high subsidence rate of nearly 30–40% (Park et al., 2019; Yao et al., 2020). Subsidence is a postoperative problem of IF, wherein the cage subsides, thus contacting the vertebral body and causing destructive effects on the vertebral endplate and even fusion failure (Marchi et al., 2013). The high subsidence risk of TLIF is induced due to its relatively smaller surface area and placement. Due to the narrow implantation channel in TLIF surgery, its size must be small; moreover, it occupies the central region of the endplate (Jin et al., 2020). The central region of the endplate has lower strength relative to its surroundings, thus causing lower stiffness (Grant et al., 2001). For the same loading, a smaller central endplate region with low stiffness caused by TLIF size behaves weakly against subsidence resistance (Palepu et al., 2018).

Although other LIFs do not have such remarkable subsidence problems, their subsidence rates raise concerns, especially PLIF and ALIF, the conventional surgeries performed in hospitals (Oh et al., 2017; Rao et al., 2017). In a new technique like OLIF, the subsidence rate can reach over 30% even with screw fixation (Zeng et al., 2018). Considering these factors, cage design poses an unavoidable challenge, especially for TLIF, and a solution for designing a cage with a relatively lower subsidence risk is expected to confer great advantages.

## Relationship between EP-vBMD and cage subsidence

**FIGURE 1:**
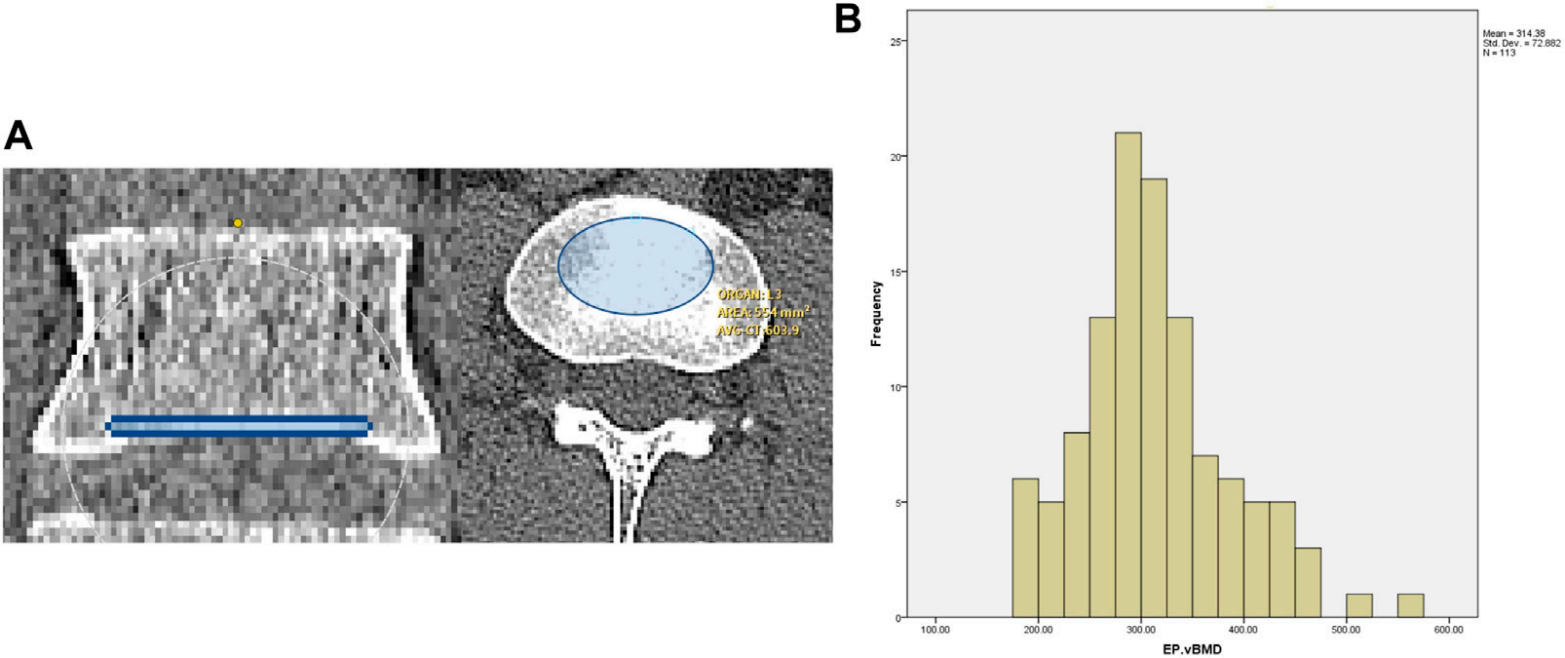
**(A)** 1.25-mm height EP-vBMD ROI **(B)** EP-vBMD distribution in this study.

In recent studies, the relationship between EP-vBMD and cage subsidence has been reported. Two studies focusing on XLIF patients have reported the relation of EP-vBMD measured by QCT with cage subsidence, however, whether EP-vBMD is an independent factor for cage subsidence, remains unclear, as ROI of 5 mm height was used for BMD measurements (Okano et al., 2020; Jones et al., 2021). MicroCT results have demonstrated that the thickness of the lumbar vertebral endplate ranges from 0.8–1 mm (Wang, 2011). In our study, an ROI of 1.25 mm height was used for EP-vBMD measurement, covering most of the endplate region but not the cortical-cancellous region (Figure 1A). Our results showed that 1.25 mm EP-vBMD was an independent predictor and its area under the curve (AUC ≈ 0.70) was higher than that for the 5 mm height cortical-cancellous region in formal work. Based on this, for patients needing IF surgery, the cortical-only 1.25 mm EP-vBMD can be a subsidence predictor and a cage customization instruction tool.

Other findings in our study showed that there may be a normal distribution for 1.25 mm EP-vBMD (Figure 1B). 78 patients (14% male and 86% female, mean age was about 62) were included in our recent study. The approximate range of the EP-vBMD was 200–500 mg/cc, and some rare cases may be outliers. Although such measurement may have limitations according to the patient population, the rough range indicated is valuable information for the application of the cage. Further studies on EP-vBMD measurement in larger patient populations are necessitated for assessing the EP-vBMD distribution.

## History of IF cage

Early development of the IF device has not focused on cage design, steel wire, rod, screw, or even direct compression methods (Virk et al., 2020). The concept of a human IF cage was indicated in 1988 by Dr. Bagby after he successfully applied this technique to horse wobbler syndrome (Wagner et al., 1979; Bagby, 1988). Subsequently, in 1998, a clinical trial showed that a bone graft with a cage (or the “Bagby busket”) could achieve a final fusion of 91%, thus increasing the treatment quality (Kuslich et al., 1998).

Material development for the cage for the improvement in IF gained substantial traction in the 1990s. Polyetheretherketone (PEEK), having an elastic modulus close to that of cortical bone was first indicated as a medical device material in 1987 (Williams et al., 1987), and was subsequently applied to cage manufacturing in a clinical trial in 1999 (Brantigan et al., 2000). The initial aim of the PEEK cage was to improve its anti-subsidence performance; however, it did not perform better for final fusion than the metallic cage showing higher modulus than bone (Seaman et al., 2017). Experiments also showed better bone cell growth with the titanic alloy surface relative to the PEEK surface (Olivares-Navarrete et al., 2012). The reason for the difficulty in bone cell integration on the PEEK surface was its hydrophobic property (low surface energy), inducing weak adhesion (biocompatibility) of cells or tissues on the implant surface and even the risk of implant rejection (Przykaza et al., 2019).

For further development of an interbody cage with better fusion and anti-subsidence performance, a metallic cage with a better bone-growth stimulating effect and anti-subsidence strategy has become a research hotspot in recent years. The elastic modulus of cortical bone is in the range of 5–20 GPa (Rho et al., 1993; Duchemin et al., 2008). The most popular metallic cage material, Ti6AL4V, has an elastic modulus of approximately 110 GPa (Niinomi, 1998), almost over 5 times the maximum cortical elastic modulus.

A porous design has been used for the metallic cage as it can reduce the elastic modulus. Comparative studies have demonstrated that the porous Ti6Al4V cage has a lower subsidence rate than the normal PEEK cage (Adl Amini et al., 2021). Additionally, a porous Ti6Al4V cage performs better in final fusion relative to the PEEK cage because its porous structure provides approachable sites for bone ingrowth, which can be directly observed from the histological results (Wu et al., 2013; McGilvray et al., 2018). Apart from fusion and anti-subsidence aspects, a recent study found that a porous Ti6Al4V cage could result in better foraminal and disc height recovery than the PEEK cage.

Further modification of the porous design of metallic cages is an obvious trend, and patient-specific customization may bring more benefits like superior fusion performance and fewer post-operative complications, thus relieving the burdens on doctors and patients.

## Biomechanically-matched EP-vBMD interbody cage development

A porous structure has recently been applied to the design of a titanium alloy cage. The porous structure is different from the mentioned hollow structure, as it contains several pores. Porous structure confers benefits on cage application including less subsidence risk and better bone growth (Park and Lehman, 2020). Reduced subsidence risk is achieved through reduced elastic modulus owing to lower density. The density-modulus relationship is universal for most materials, its mathematical expression is shown below (Murr, 2020).

$$\frac{E}{E_{s}}={a\left(\frac{\rho }{\rho_{s}}\right)}^{b}$$

The equation above implies that there is a power law between material density and elastic modulus, E means porous material elastic modulus, $E_{s}$ means solid-state material elastic modulus, ρ means porous material density, $\rho_{s}$ means solid-state material density, a and b are constants. The density ratio can be directly translated to the porosity which indicates the porous volume percentage. Therefore, by changing the porosity, the material’s modulus can be altered.

The porosity-modulus strategy enables better performance of the titanium alloy cage, and some studies have demonstrated that porous titanium alloy cage has relatively lower subsidence risk and better fusion performance than PEEK cage, owing to better osteointegration (Wu et al., 2013; Adl Amini et al., 2021). Apart from the osteointegration benefit, an improvement in the biomechanical aspect is likely as the subsidence becomes evitable in osteoporotic or osteopenia patients (Hasegawa et al., 2001; Wu et al., 2022). Differences in elastic moduli between the implant and bone cause stress shielding, finally leading to the weakening of the bone (Murr, 2020). In cases of osteoporotic or osteopenia, a porous cage with one standard modulus may not a suitable choice due to the differences in the moduli (Li et al., 2019; Pan et al., 2021).

Accordingly, a patient-specific strategy for better subsidence prevention could be indicated based on the vBMD-subsidence relationship, as evidenced by a patient-specific biomechanically matched interbody cage demonstrated in this study. By calculating the compressive elastic modulus of a specific QCT vBMD measured on the spinal endplate (EP-vBMD-E), it was possible to customize Ti6Al4V porous alloy with a specific EP-vBMD-E.

Although the relationship between spinal endplate cortical QCT-BMD and its compressive elastic modulus is undetermined, some research has summarized relevant theories covering most of the EP-vBMD region (200–500 mg/cc) (Keyak et al., 1994; Morgan et al., 2003). By comparing the calculated results with other published measurements (Kaneko et al., 2003; Bayraktar et al., 2004; Duchemin et al., 2008), the validity of mentioned theories on calculating compressive elastic modulus of endplate cortical bone was confirmed.

In this study on EP-vBMD, the rough minimum and maximum EP-vBMD values were 200 mg/cc and 500 mg/cc, respectively. Considering the diagnostic standard for orthopedic diseases like osteoporosis, a primary EP-vBMD cage grading scheme can cover 3 grades, therefore, apart from the characteristic values of high EP-vBMD (500 mg/cc) and low EP-vBMD (200 mg/cc), an intermediate EP-vBMD can be implemented into the grading scheme at 350 mg/cc, the average of low and high EP-vBMD values.

**FIGURE 2:**
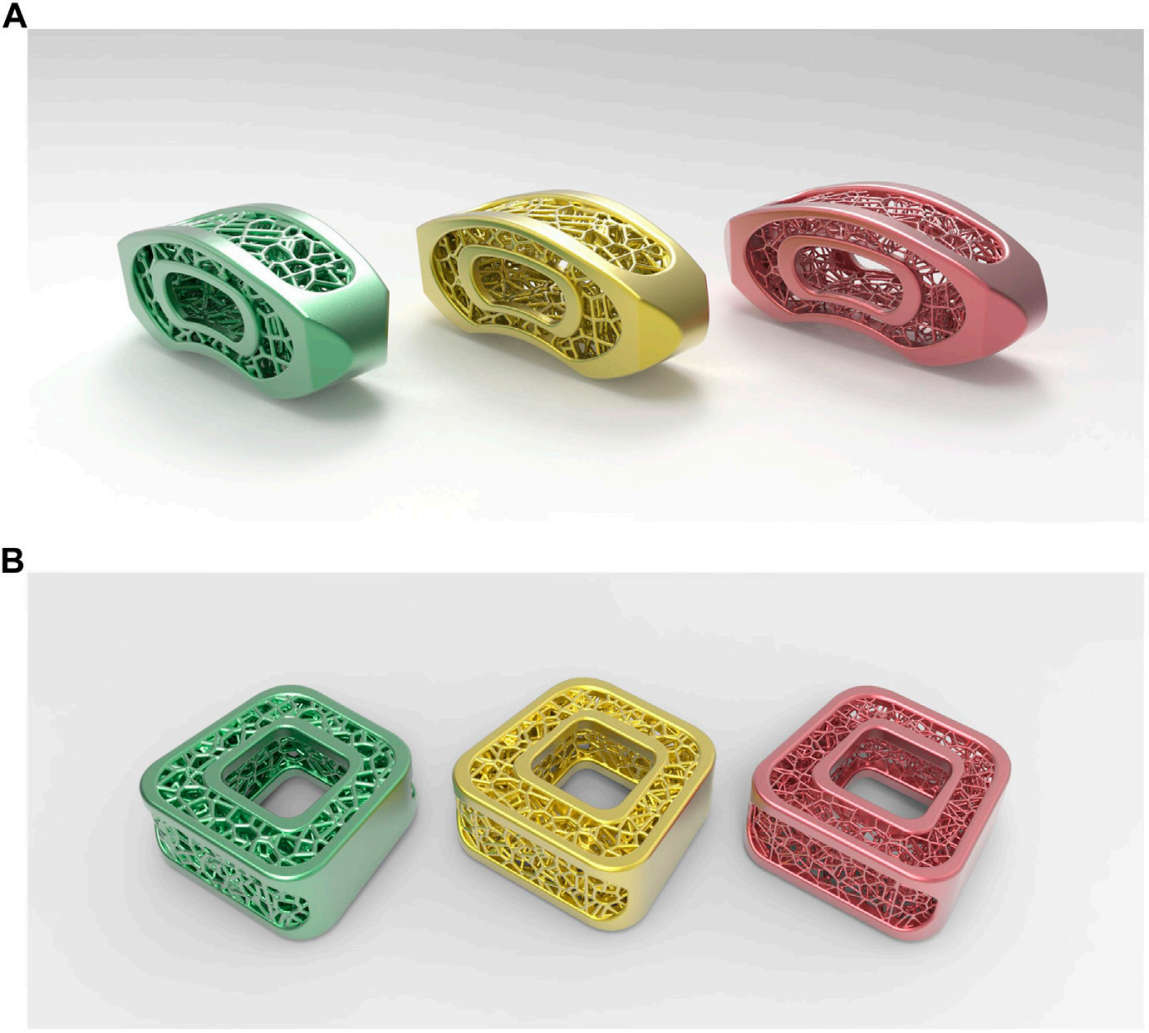
High-to-low endplate vBMD cages for lumbar **(A)** and cervical **(B)** fusion.

Accordingly, a primary 3-grade scheme for EP-vBMD-E cage customization is indicated herein, namely low EP-vBMD cage (LEDC), middle EP-vBMD cage (MEDC), and high EP-vBMD cage (HEDC). The mentioned cages have elastic moduli close to 200 mg/cc, 350 mg/cc, and 500 mg/cc EP-vBMD, correspondingly, which can be confirmed by a convergent finite element model in LIF cage design (Figure 2A). 0.1-mm tetrahedral mesh was applied in LIF cage finite element analysis, a uniaxial compressive loading was applied on superior contacting surface, the inferior contacting surface was fixed in all directions. The same design could also be applied to the cervical interbody cage design (Figure 2B).

Averages between low, middle, and high EP-vBMD characteristic values are calculated for the specific classification ranges. Accordingly, EP-vBMD> 425 mg/cc, 275 mg/cc ≥ EP-vBMD≥425 mg/cc, and 275 mg/cc ≥ EP-vBMD may indicate high, middle, and low EP-vBMD ranges, suggesting the utility of HEDC, MEDC, or LEDC accordingly. The images below represent the 3-grade EP-vBMD-E cage customization strategy (Figures 2A,B).

For some rare cases with EP-vBMD higher than 500 or less than 200, it is possible to further customize as there is a relationship between the cage porosity and cage modulus, and such customization could solve the above-mentioned issue and the design can be manufactured as industrial selective laser melting (SLM) can achieve 0.1 mm wall thickness (on a typical Ti6Al4V material) (Wu et al., 2020), smaller than the minimum dimension of our porous cage unit rod. Relevant animal validation, biomechanical experiments, and clinical testing will be proposed in future work for boosting its clinical translatability.

## Discussion

Porous design on the metallic interbody cage is an obvious trend, especially for better fusion and anti-subsidence effect. Our EP-vBMD patient-specific interbody design may bring more benefits. If there is a special need from doctors or patients, direct customization based on the porosity-modulus theory can be achieved, not only limited to patients whose EP-vBMD exceeds 500 mg/cc or less than 200 mg/cc. The detailed porosity-modulus relationship of our novel cage design will be assessed in further research. Several studies have focused on the porosity-modulus of scaffolds and similar testing methods can be applied to our cage design (Liang et al., 2019; Du et al., 2020). Existing mechanical research mainly focuses on finite element analysis (FEA) of the cage design, which not only demonstrates the simulated porosity-modulus relationship but also the contour-result variation. The significance of contour results is in illustrating more details like the stress/strain distribution, which may save experimental costs during cage development. Another benefit of the FEA validation on the porosity-customized cage is that if primary validation results can confirm the reliability of the simulation, its contour results may be directly used as the official evaluation parameter, thus saving time and money for further complex experiments.

There are also some possible improvements to the novel cage customization design. The endplate area is inhomogeneous, and the density of the surrounding area of the endplate is higher than that of the inner area (Segami et al., 2021). Theoretically, in cases of a relatively larger-size cage like the XLIF cage, a localized EP-vBMD design may increase its compatibility with a specific patient. The EP-vBMD-E theory is not a direct methodology, and more experiments are necessitated to obtain a novel cage design. According to our findings on the effect of endplate thickness on EP-vBMD ROI, some mechanical experiments focused on a 1 mm spinal endplate sample will be conducted in the future, which is expected to be challenging and worthwhile.

Some advanced topics like biomechanical interaction between the porous cage and patient-specific spine will also be considered. *In vivo* experiments have some limitations. *In vivo* mechanical testing on cage-spine interaction caused by loading constraints remains a farfetched possibility. A previous study on skin explored the feasibility of *in vivo* mechanical experiments (Pailler-Mattei et al., 2008). FEA has some advantages like contour results and a patient-specific reconstruction model includes patient-specific EP-vBMD, which may be the primary choice for assessing the interaction between cage and spine segments. Unlike *in vivo* animal validation, it focuses on biomechanical results including the stress/strain distribution on the cage-spine contacting area. Moreover, a patient-specific spine-cage model with a material setting based on mentioned experiments and simulation could provide accurate results on cage-spine biomechanical simulation. Like FEA of cage porosity grade, this may serve as a low-cost practical cage design instruction tool for final clinical determination after validation.

In summary, the cage subsidence problem as an inevitable postoperative risk necessitates a biomechanical solution. Through EP-vBMD measured by phantomless QCT, a theory that connects EP-vBMD, endplate elastic modulus, and cage porosity was established. Three-grade biomechanically EP-vBMD cage design may be a suitable primary strategy. A direct patient-specific EP-vBMD cage customization may be necessary for clinical decisions, which could be facilitated through a methodology based on simulation and experiments.

## Data Availability

The raw data supporting the conclusions of this article will be made available by the authors, without undue reservation.

## Abbreviations

PL-QCT, phantomless quantitative computed tomography; DXA, dual-energy x-ray absorptiometry; IF, interbody fusion; ALIF, anterior lumbar interbody fusion; PLIF, posterior lumbar interbody fusion; LLIF, lateral lumbar interbody fusion; TLIF, transforaminal lumbar interbody fusion; OLIF, oblique lumbar interbody fusion; EP-vBMD, endplate volumetric bone mineral density; ROI, region of interest; PEEK, Polyether Ether Ketone; EP-vBMD-E, compressive elastic modulus of a specific QCT vBMD measured on spinal endplate; LEDC, low EP-vBMD cage; MEDC, middle EP-vBMD cage; HEDC, high EP-vBMD cage; SLM, selective laser melting.
